# Nutritional, Anti‐Nutritional and Mineral Contents of Selected Wild Edible Plants in Ethiopia: *Mussaenda arcuata*, *Celosia trigyna,* and *Pteridium aquilinum*

**DOI:** 10.1002/fsn3.70530

**Published:** 2025-06-27

**Authors:** Tamene Daba Rumicha, Sirawdink Fikreyesus Forsido, Yetenayet Bekele Tola, Abebe Yimer, Chala G. Kuyu, Tilahun A. Teka, Amira Gidi

**Affiliations:** ^1^ Department of Postharvest Management College of Agriculture and Veterinary Medicine, Jimma University Jimma Ethiopia; ^2^ Department of Food and Nutritional Sciences, Faculty of Agriculture Wollega University Nekemte Ethiopia

**Keywords:** anti‐nutritional factor, bioavailability, molar ratio, proximate composition, wild edible plants

## Abstract

Despite the presence of several wild edible plants in Ethiopia, communities across the country continue to face serious food and nutrition challenges. The study was carried out to evaluate nutritional composition, anti‐nutritional factor content, mineral composition, and mineral bioavailability in *Mussaenda arcuata fruits*, *Celosia trigyna leaves*, and *Pteridium aquilinum fronds* consumed in the southwestern parts of Ethiopia and reported as dry weight basis. The study result revealed that they are rich in crude fiber content, which may play a role in reducing serum cholesterol and blood pressure and the risk of coronary heart disease. The leaves of *Celosia trigyna* and *Pteridium aquilinum* were wild edible plants rich in protein, ranging from 29.89 to 31.76 g/100 g. The *Mussaenda arcuata fruit* was characterized by a higher crude fat (6.47 g/100 g) and a higher total energy content (332.72 Kcal/100 g). The study also found that minerals such as K, Na, Ca, Mg, P, Zn, and Fe were present at optimal and safe levels, with their molar ratios falling within acceptable limits. The contents of the anti‐nutritional factors (condensed tannins, phytate, and oxalate) were below the maximum acceptable limits and did not affect the bioavailability of calcium, iron, and zinc. Therefore, the fruits of *Mussaenda arcuata*, *Celosia trigyna* leaves, and fronds of *Pteridium aquilinum* are important wild edible plants that can contribute to improving food and nutritional security in the region.

## Graphical abstract

Mussaenda arcuate fruit has high energy (332.72 kcal/100 g) and crude fat, ideal for caloric supplementation. *Pteridium aquilinum* fronds and *Celosia trigyna* leaves are rich in protein (29.89–31.76/100 g), supporting dietary protein needs. All species contain essential minerals (K, Ca, Mg, P, Fe, Zn) in bioavailable forms, with anti‐nutritional factors below safe limits.

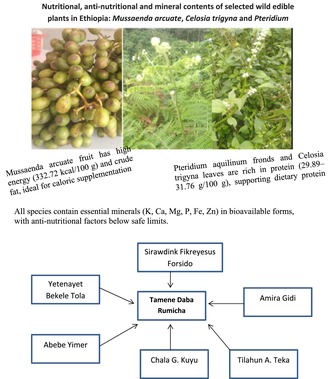

## 1 Introduction

Wild edible plants represent a minor contribution to family meals that are potentially essential nutrients and cultural resources for local people worldwide. They often contain higher amounts of nutrients and bioactive compounds than many cultivated species, especially those that have been under cultivation for many generations (Fentahun and Hager 2009; Martins et al. 2011; Heinrich et al. 2016; Trumbo et al. 2002).

Food and nutrition security needs to be guaranteed in the context of biodiversity, an important asset to domesticate new crops or improve the quality of traditional crop plants (FAO 2010; Johns and Eyzaguirre 2007; Dhar et al. 2012). By 2025, Ethiopia is still facing a severe food security crisis driven by a combination of conflict, climate shocks, economic instability, and critical funding shortages. More than 10 million people are currently suffering from hunger and malnutrition, with more than 3 million internally displaced persons (IDPs) due to conflict and climatic disruptions such as droughts and floods (WFP 2025). In this context, underutilized wild edible plants offer promising, locally available solutions to enhance dietary diversity and nutritional intake, particularly in rural and resource‐limited areas (Rumicha et al. 2025)

Wild edible plants (WEPs) are crucial in improving food security and resilience among rural and marginalized communities in Ethiopia. These plants serve as crucial food sources during periods of drought, crop failure, and seasonal shortages when conventional agriculture often falls short (Guinand and Lemessa 2001). Nutritionally, WEPs are rich in essential vitamins, minerals, fiber, and antioxidants, helping to diversify diets and address common micronutrient deficiencies found in staple‐based diets (Baye 2014). In particular, they provide significant amounts of protein, vitamin B_2_ (riboflavin), and vitamin C, which are critical to maintaining health and preventing malnutrition (Fentahun and Hager 2009). Beyond their nutritional value, WEPs offer economic benefits. Many species are traded in local markets, contributing to household income and supporting rural livelihoods (Fentahun and Hager 2009). Their use is also deeply rooted in indigenous knowledge systems, which promote sustainable harvesting and the conservation of biodiversity (Balemie and Kebebew 2006). Importantly, WEPs are well adapted to Ethiopia's diverse and often harsh environments. Their resilience to climate variability makes them important assets for agroecological sustainability, especially as communities face the growing challenges of climate change and land degradation.

Despite their potential, the scientific exploration of WEPs in Ethiopia remains limited in scope. Most existing studies have focused primarily on a narrow range of species, often emphasizing only on their ethnobotanical or culinary uses, with less attention given to their detailed nutritional and mineral profiles (Kibar and Kibar 2017). As a result, there is a significant knowledge gap about the full nutritional value of many wild species that are regularly consumed by local populations. This underrepresentation in the scientific literature impedes its potential inclusion in national nutrition strategies and policy frameworks to combat malnutrition.

In addition, WEPs can also contain anti‐nutritional factors such as phytates, oxalates, and tannins, which can interfere with the absorption and bioavailability of key nutrients, particularly minerals such as iron, zinc, and calcium. Therefore, it is important to quantify their nutritional and mineral content, evaluate the presence of these anti‐nutritional compounds, and assess their potential impact on nutrient bioavailability. The present study seeks to address these research gaps by investigating the nutritional composition, anti‐nutritional factors, mineral content, and mineral bioavailability of three underutilized wild edible plant species: *Mussaenda arcuata, Celosia trigyna*, and *Pteridium aquilinum* , which are commonly consumed in southern Ethiopia. By generating comprehensive nutritional data on these species, the study aims to contribute to a broader understanding of their role in promoting food and nutritional security and to support efforts to integrate wild food resources into sustainable dietary systems.

## 2 Materials and Methods

### 2.1 Description of the Study Sites

**FIGURE 1:**
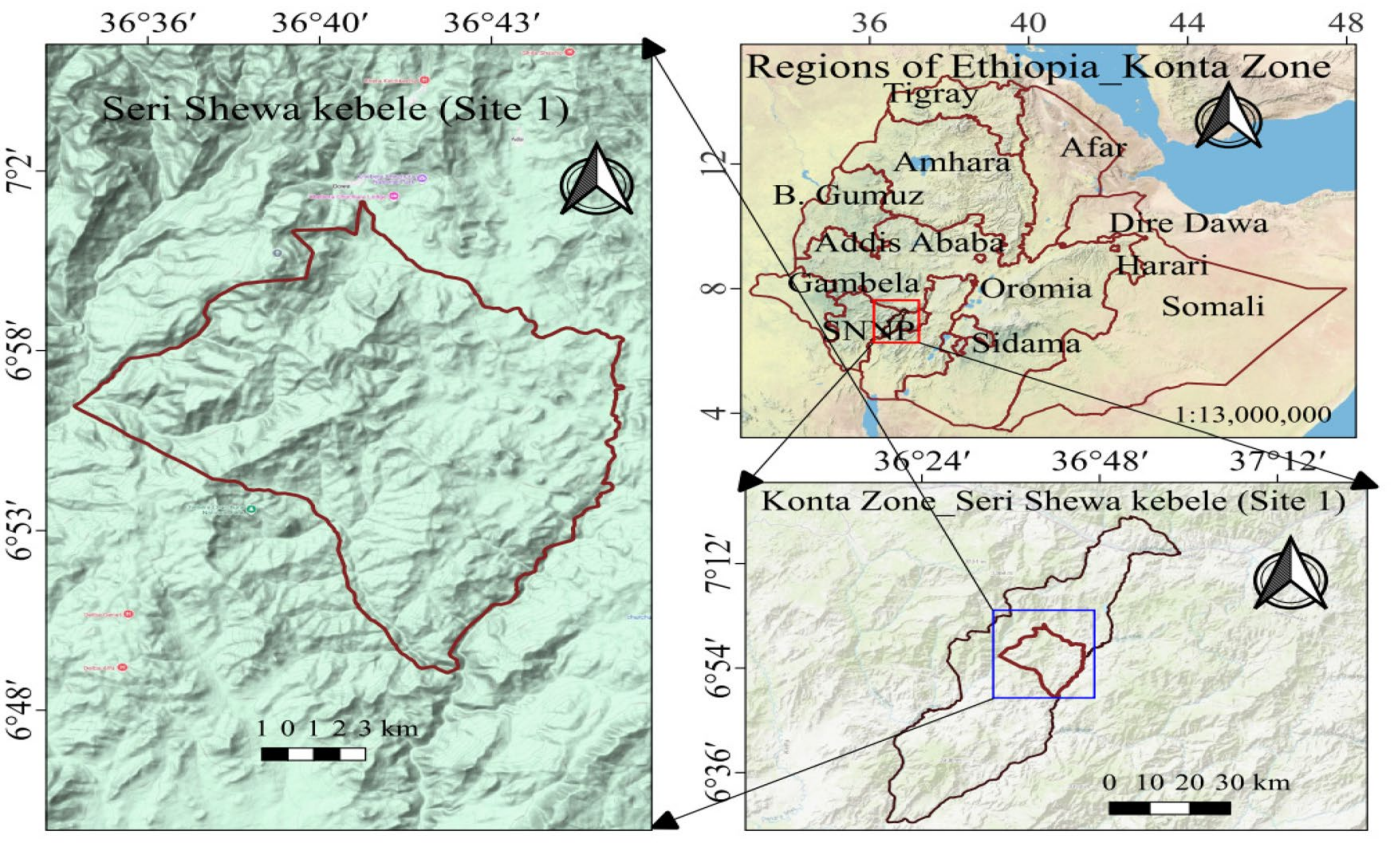
Map of study site 1 (Seri Shewa Kebele).

**FIGURE 2:**
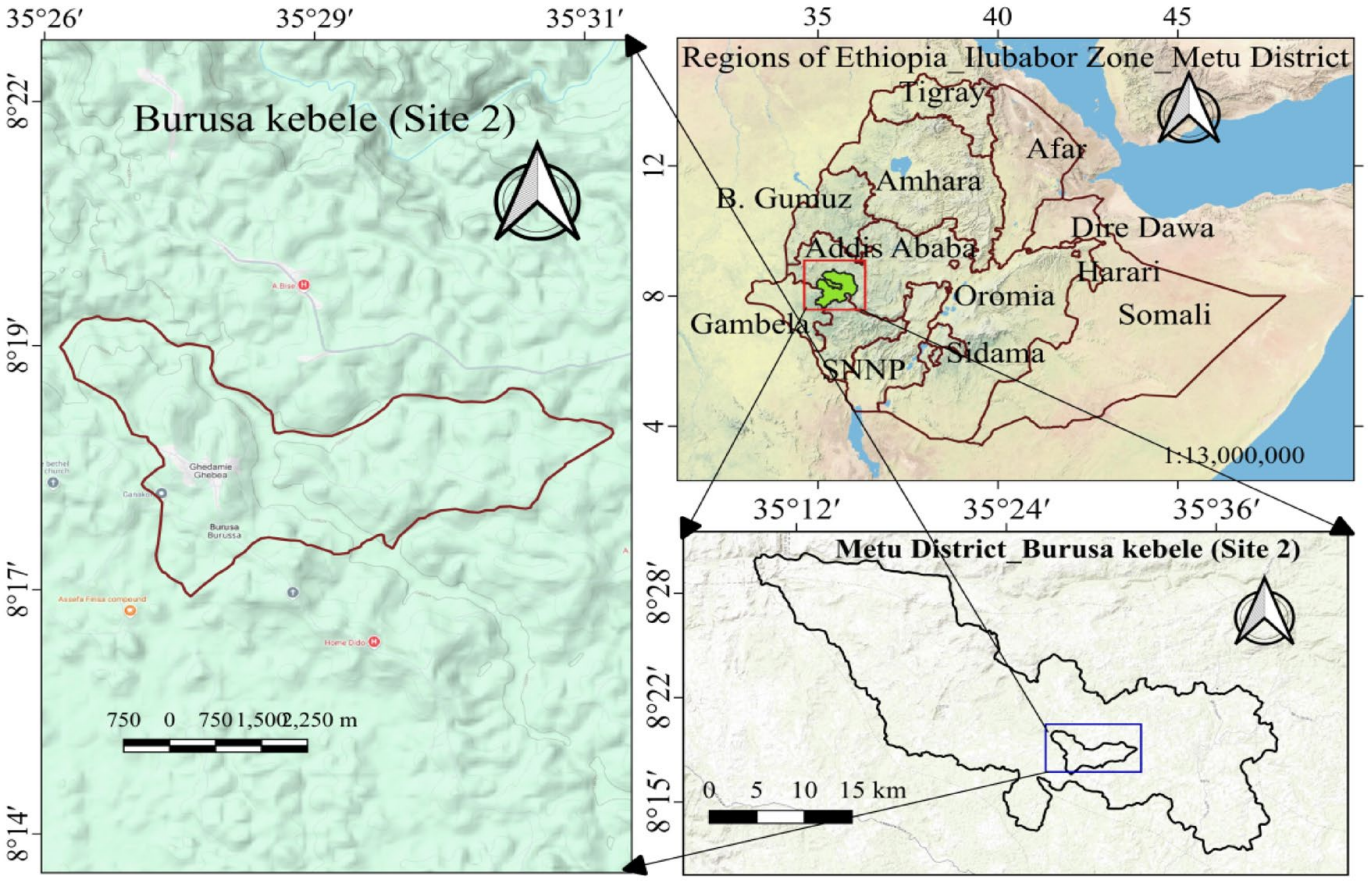
Map of the study site 2 (Burusa Kebele).

The samples for the study were collected from two study sites, namely the Chebera Churchura National Park area of the Konta Zone and the Ilubabor zone Figures 1 and 2. Study sites were selected based on potential sources of availability of wild edible plants. Chebera Churchura National Park is located between the Dawuro and Konta zones in southern Ethiopia and is administered by the two zones. The park is 468 km from Addis Ababa and 133 km from Jimma City. The Ilubabor zone was located in the Oromia regional state and has a wide coverage of natural forests. Metu, a city in the zone, was found 537 km away from the capital city of the country (Addis Ababa) and 254 km away from Jimma City.

### 2.2 Sample Collection, Identification, and Preparation

The study sites were purposely selected based on an ethnobotanical study of wild edible plants (WEPs) in Ethiopia. The Konta and Ilubabor zones were chosen because of their known reliance on WEPs. Within these zones, Konta special Woreda and Metu Woreda were identified through consultations with local officials as areas where communities widely use WEPs. Further discussions with the respective Woreda Agricultural Offices helped identify key kebeles: Seri Shewa Kebele in Konta special Woreda and Burrusa Kebele in Metu Woreda, where WEPs are particularly prominent.

In each kebele, local administrators and development agents assisted in the purposeful selection of 12 key knowledgeable informants from community members who frequently use and benefit from WEPs. These informants were first individually interviewed using a structured questionnaire to document commonly consumed WEPs, including plant parts used, habitat, modes of consumption, seasonal availability, preparation methods, and local uses. Subsequently, group interviews were conducted with the same informants to validate and deepen the information gathered.

From the list of identified WEPs, key species were selected for further study based on predefined criteria: multipurpose use (both as food and medicine), novelty (species with limited or no prior nutritional and pharmacological studies), availability (accessible when needed) and ease of domestication. On the basis of these criteria, *Mussaenda arcuata fruit* from Seri shewa Kebele and *Celosia trigyna* and *Pteridium aquilinum* from Burrusa Kebele were selected and collected for further analysis.

The wild edible plant samples used for botanical identification were collected from the field with the help of a botanist from Addis Ababa University. The samples were then submitted to the National Herbarium at Addis Ababa University for formal taxonomic identification. Mr. Melaku Wendafrash, a professional botanist at the institution, performed the identification. Consequently, wild edible *Mussaenda arcuata* (voucher number AY‐04), *Celosia trigyna* (voucher number AY‐13), and *Pteridium aquilinum* (voucher number AY‐67) were accurately identified and confirmed.

**FIGURE 3:**
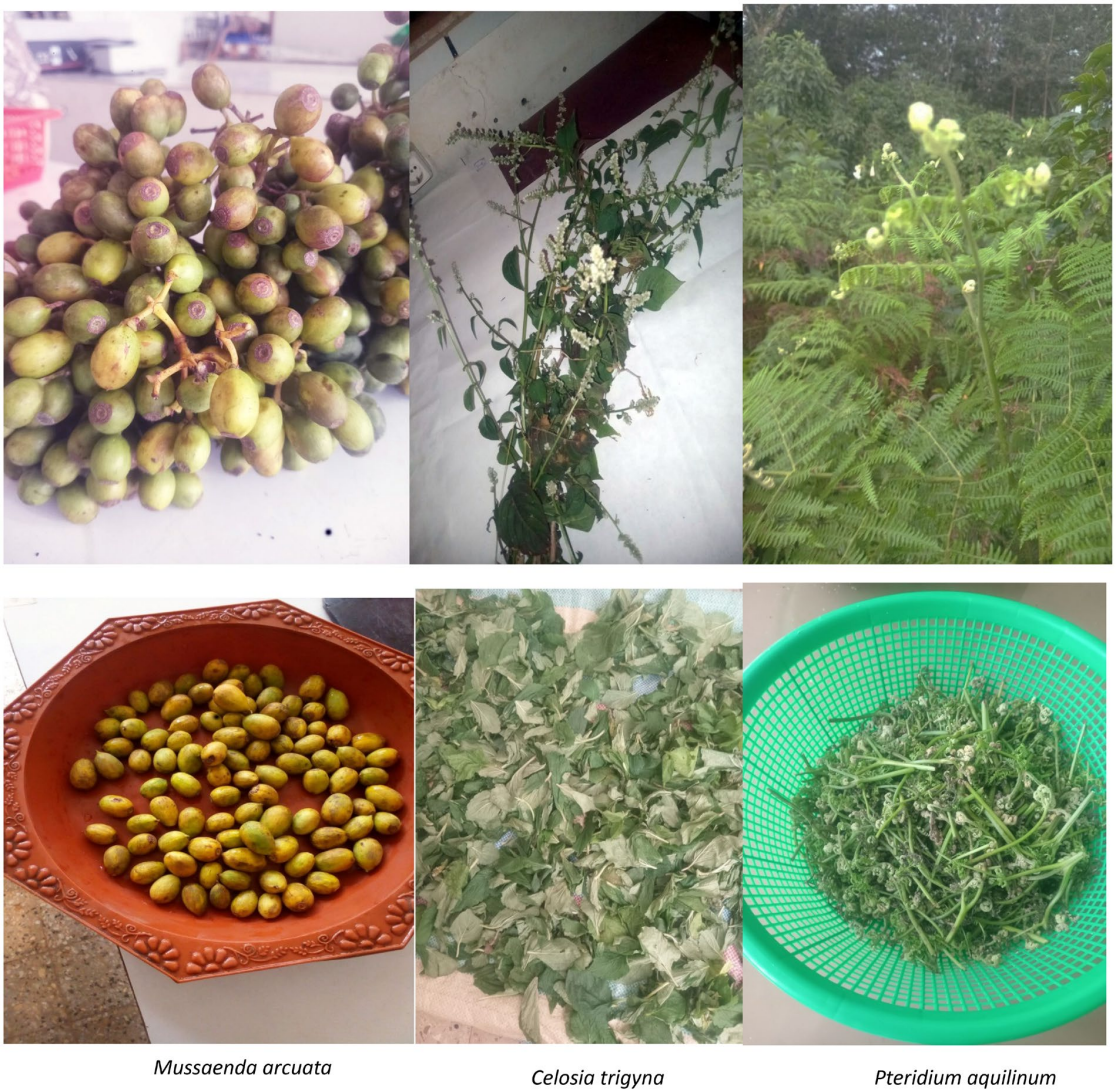
Study species of wild edible plants.

The mature and ripened fruit required for analysis was collected and transported to the Jimma University College of Agriculture and Veterinary Medicine Post Harvest Management Laboratory with an icebox. Furthermore, clean and healthy leaves of *Celosia trigyna* and healthy fiddlehead (frond) of *Pteridium aquilinum* were collected and transported to the Jimma University College of Agriculture and Veterinary Medicine Post Harvest Management Laboratory with an icebox. The studied wild edible plant species are illustrated in Figure 3. The collected samples were stored in a freezer at −4°C until analysis and sample preparation.

Fresh and healthy fruit, leaf, and fronds of collected WEPs were sorted, cleaned, and trimmed prior to sample preparation for analysis. Samples were prepared according to standard procedures. The samples were dried in drying ovens (DHG‐9203 A, Shanghai, China) kept at 45°C for 18 h to preserve heat sensitive nutrients such as vitamin C and bioactive compounds such as total phenolic content, total flavonoids and β‐carotene (Addis et al. 2009).

### 2.3 Determination of the Proximate Composition

#### 2.3.1 Moisture Content Determination

Moisture content was determined by the oven drying method as AOAC (2005) method 925.10. An empty drying crucible was dried in the oven at 105°C for 1 h, cooled in a desiccator, and weighed (*W*_1_). A 1 g fresh wild edible plant was weighed and poured into a precleaned and weighed drying crucible (*W*_2_). The drying crucible with the sample was then placed in an oven dryer (DHG‐9203 A, Shanghai, China) and allowed to dry at 105°C until a constant weight was reached, cooled in a desiccator, and finally weighed (*W*_3_). The weight loss after drying was determined as a percentage of the moisture content using the equation:

(1) $$\text{Moisture content}\;\left(\%\right)=\left(\frac{W_{3}-W_{1}}{W_{2}}\right)\times 100$$

Where: *W*_1_ is the weight of the dried crucible, *W*_2_ is the weight of the sample before drying, *W*_3_ is the weight of the sample and the crucible after drying

#### 2.3.2 Crude Fat Determination

The crude fat contents of samples were analyzed using a Soxhlet extractor (Soxhlet Fat Analyzer, Zhejiang, China) as described in AOAC (2005) Method 920.39, with some modifications. Approximately 1 g (Ws) of dried sample was weighed and placed in a precleaned thimble. The thimble was inserted into a Soxhlet apparatus, and extraction was carried out using n‐hexane as the solvent at a solvent‐to‐sample ratio of 10:1 for digestion at 120°C for 8 h. Hexane was used as a solvent for fat extraction due to its low boiling temperature, ease of oil recovery, and excellent solubilizing ability. As a modification to the standard AOAC procedure, instead of allowing the solvent to evaporate through conventional heating, the solvent was removed from the lipid extract under reduced pressure using a rotary evaporator (Buchi Rotavapor R‐300) at 40°C. This modification was used to minimize the degradation or loss of heat‐sensitive lipid components and to improve the efficiency and safety of solvent recovery. The remaining lipid residue was then dried in a hot air oven at 105°C for 30 min to remove any residual solvent, cooled in a desiccator, and weighed to a constant mass. The crude fat content was expressed as a percentage of the dry weight of the sample. The samples were cooled in a desiccator and weighed (*W*_f_). Crude fat was calculated with the formula;

(2) $$\text{Crude}\;\mathrm{fat}\;\left(\%\right)=\left(\frac{W_{f}}{W_{s}}\right)\times 100$$

Where: *W*_f_ (weight of fat) = weight of thimble after extraction‐eight of thimble before extraction, *W*_s_ = weight of samples (db).

#### 2.3.3 Determination of the Crude Protein

The crude protein content of the samples was determined using the Kjeldahl method as described in AOAC (2005), Method 920.87, with the aid of an automated Kjeldahl apparatus (UDK 159, VELP Scientific, Nemko, USA, Italy). Approximately 1 g of the sample was measured with a digital scale and transferred into a Kjeldahl digestion tube. To facilitate digestion, 5 g of K_2_SO_4_ and 2 g of CuSO_4_ catalysts were weighed using an electronic balance and added to each simple digestion tube containing the sample. Then, 25 mL of concentrated H_2_SO_4_ was poured into each digester tube containing the sample and catalyst solution. The digestion was carried out at 420°C for 2 h, cooled to ambient temperature in the digester, and formed an ammonium sulfate solution. After the sample had cooled, the digestion tube was inserted into the distillation and titration unit. The sample solution was treated with NaOH and boiled to convert NH_4_ to NH_3_. Then, the ammonia in the sample was trapped in a 4% boric acid solution containing 7 mL of methyl red and 10 mL of bromocresol green, and the instrument was automatically titrated with 0.2 N HCl. A 0.2 N HCl solution used for titration in the determination of the crude protein was standardized with a primary base (sodium carbonate) prior to use to ensure its accuracy in determining the nitrogen content. After the distillation and titration process, the instrument displayed the total amount of nitrogen and a conversion factor of 6.25 was used.

#### 2.3.4 Determination of Crude Fiber

The crude fiber content was analyzed gravimetrically using the modified AOAC (2005) method 985.29 with a fiber analyzer (SLQ‐6A, Shanghai, China). A 0.5 g sample was weighed with an analytical balance (*W*_3_). The sample was boiled in 1.25% H_2_SO_4_ in a beaker for 30 min and then hydrolyzed for another 30 min with 1.25% NaOH. The sample was rinsed with hot water and acetone and dried at 105°C for 1 h until a constant weight was obtained, which was cooled in a desiccator and weighed (*W*_1_). The sample residue was re‐ignited in a muffle furnace kept at 550°C for 3 h, cooled in a desiccator and weighed again (*W*_2_). The crude fiber content was determined using the equation:

(3) $$\text{Crude fiber}\;\left(\%\right)=\left(\frac{W_{1}-W_{2}}{W_{3}}\right)\times 100$$

Where: *W*_1_ = weight of the sample washed with acid and base after drying at 105°C for 1 h. *W*_2_ = weight of the washed and dried sample after being ignited at 550°C for 3 h. *W*_3_ = sample weight before drying (db).

#### 2.3.5 Determination of Ash

The ash content of the sample was analyzed by igniting the sample in a muffle furnace according to AOAC (2005) method 923.03. The washed and dried crucible was weighed with an analytical balance (*W*_1_). A 2 g sample was weighed and poured into the weighed crucible (*W*_3_). The sample was ignited at a temperature of 550°C in a muffle furnace (Nabertherm, D‐6072 Dreieich, Germany) for 4 h until a gray‐white color developed. Lastly, the sample was cooled in a desiccator for an hour and weighed (*W*_2_). The ash content can be determined with the equation:

(4) $$\mathrm{Ash}\;\left(\%\right)=\left(\frac{W_{2}-W_{1}}{W_{3}}\right)\times 100$$

Where: *W*_1_ = weight of washed and dried crucible, *W*_2_ = weight of dried sample with crucible, *W*_3_ = weight of sample before drying

#### 2.3.6 Determination of Carbohydrate

Carbohydrate (%) was determined by subtracting the sum of the ash, crude fat, crude fiber, and crude protein values from 100.

### 2.4 Calculation of Total Energy Content

The calorific value (kcal/100 g sample) was calculated using Atwater factors of 9× for crude fat and 4× for crude protein and total carbohydrate.

### 2.5 Determination of Mineral Content

The mineral content was determined by microwave‐induced plasma atomic emission spectrometry (MP‐AES) following the AOAC (2016) method 984.27 after carbonization on a heating plate and dry ashing. 3 g of WEPs flour was weighed, dried in an oven at 105°C overnight, and cooled to room temperature in a desiccator. The dried sample was burned in a muffle furnace (Nabertherm, D‐6072 Dreieich, Germany) at 450°C for 4 h. The sample was cooled in a closed oven. The ashed sample was transferred to a 200 mL flask with 20 mL of 20% HNO_3_, heated to near boiling on a hot plate for 30 min while intermittently stirring with a glass rod and allowed to cool. The sample was filtered using Whatman number 4 filter paper and made up to a volume of 100 mL with distilled water. A series of standard solutions containing all elements of interest was prepared. Blank samples for mineral analysis were prepared from distilled water mixed with the reagents under the same conditions as the treated samples. The sample aliquot of minerals was determined by microwave‐induced plasma atomic emission spectrometry (MP‐AES) using plasma nitrogen (N_2_) gas. Each mineral element of the sample was measured at the required emission wavelength for calcium (393.366 nm), potassium (766.491 nm), sodium (588.995 nm), magnesium (285.213 nm), iron (371.993 nm), zinc (213.857 nm), and phosphorus (410 nm). The respective minerals were determined using the following equation:

(5) $$\text{Mineral Concentration}\;\left(\frac{\mathrm{mg}}{100\;g}\right)=\frac{\left(a-b\right)\times V\times D}{10\times \mathrm{Ws}}$$

Where: *a* = concentration in ppm (mg/L) of the sample, *b* = concentration in ppm of the blank solution, *V* = volume in mL of extract,*D* = dilution factor (50 mL for Na, K, Ca, Mg, Fe, Zn) and 100 mL for P. Ws = sample weight in g and10 = conversion factor

### 2.6 Determination of Mineral Ratio

The mineral ratios in the wild edible plants were determined by first converting the mass of each mineral to moles, using their respective atomic weights (K = 39 g/mol, Na = 23 g/mol, Ca = 40 g/mol, Mg = 24 g/mol, *p* = 31 g/mol, Fe = 56 g/mol, Zn = 65 g/mol). The molar ratio between two minerals was then calculated by dividing the number of moles of the first mineral by the number of moles of the second mineral (Jacob et al. 2015).

### 2.7 Determination of Antinutrient Content

#### 2.7.1 Determination of the Oxalate Content

The oxalate contents in food samples determined according to the AOAC (2000) methods with minor modifications. 2 g of the sample was mixed with 190 mL distilled water and 10 mL of 6 M HCl and digested at 100°C for 1 h. The digested sample was cooled, diluted to 250 mL, and filtered with filter paper. 125 mL of the filtrate was poured into the beaker, mixed with four drops of methyl red indicator, and titrated with concentrated NH_4_OH until the test solution changed from salmon pink to a faint yellow color (PH 4–4.5). The 125 mL portion of the solution was heated to 90°C, cooled, and filtered to remove the precipitate of ferrous ions. The filtrate was also heated to 90°C, adding 10 mL of 5% CaCl_2_ solution with constant stirring. After heating and cooling, the filtrate was left to stand overnight in a refrigerator at 5°C. The decanted and precipitated supernatant was dissolved in a 10 mL 20% (v/v) H_2_SO4 solution. The total filtrate from digestion of the 2 g sample was diluted to 300 mL; 125 mL of filtrate aliquots were heated to near boiling and titrated with a standard solution of 0.05 M KMnO_4_ to a faint pink color change, which persisted for 30 s. The calcium oxalate content was expressed as the calcium oxalate equivalent and calculated using the equation:

(6) $$\text{Oxalate}\;\left(\frac{\mathrm{mg}}{100g}\right)=\frac{T\times \mathrm{Vme}\times \mathrm{DF}\times 10^{5}}{\mathrm{ME}\times \mathrm{MF}}$$

Where:*T* is the KMnO_4_ titer (mL), Vme is volume‐mass equivalent (i.e., 1 cm^3^ of 0.1 N KMnO_4_ solution is equivalent to 0.006303 g anhydrous oxalic acid), DF is the dilution factor, *r* (2.4): it is the total volume of the filtrate (300 mL) divided by the aliquot used for titration (125 mL), ME is the molar equivalent of KMnO_4_ in oxalate (KMnO_4_ redox reaction), which is 126/3 = 63, and MF is the mass of flour used.

#### 2.7.2 Determination of Phytate Content

The phytate content was analyzed according to the methods described in Vaintraub and Lapteva (1988). A 0.5 g sample was extracted with 10 mL of 2.4% HCl using a mechanical shaker (Hy‐2(C), Shanghai, China) for 1 h at room temperature. The extract was centrifuged at 3000 rpm for 30 min (Sigma 2–16 KC, UK). The clear supernatant was used for phytate determination. 1 mL of Wade's reagent (containing 0.03% solution of FeCl_3_ 6H_2_O and 0.3% sulfosalicylic acid in water) was added to 3 mL of the sample solution (supernatant), and the mixture was vortexed for 5 s. The absorbance of the sample solutions was measured at 500 nm using a UV–VIS spectrophotometer (721 Visible Spectrophotometer, Shanghai, China). A series of phytic acid sodium salt standard solutions was prepared containing 0‐150 μg/mL phytic acid (analytical grade sodium phytate) in 0.2 N HCl. 1 mL of Wade's reagent was added to each tube, and the solution was vortexed for 5 s. The mixtures were centrifuged for 10 min and the absorbance of the standard solutions was measured at 500 nm using a UV‐spectrophotometer. Deionized water was used as a blank for the standard solutions and samples. The phytate content was determined from a standard curve of the sodium salt of phytic acid, and the result was reported as mg/100 g.

A standard curve was derived from the absorbance versus concentration of sodium phytate standard. The phytate content was calculated using the following formula, and the result was reported in mg/100 g.

(7) $$\text{Phytate}\;\left(\frac{\mathrm{mg}}{100g}\right)=\frac{\left[\left(\mathrm{AbS}-\mathrm{AbB}\right)-\text{Intercept}\right]\times 10}{\text{slope}\times W\times 3}$$

Where:*W* = weight of the sample in grams. AbS = sample absorbance, AbB = blank absorbance, 10 = conversion factor from μg/mL to mg/100 g, 3 = volume of aliquot

#### 2.7.3 Determination of Condensed Tannin Content

Condensed tannin was analyzed using the methods stated by Maxson (1972). A 2 g dried sample was mixed with 10 mL of 1% HCl in methanol solution in a 50 mL Falcon tube, followed by shaking the solution on a mechanical shaker (Hy‐2(C), Shanghai, China) at room temperature for 24 h. The solution was centrifuged using a centrifuge machine (Sigma Laborzentrifugen, 2–16 KC, Germany) at 1000 rpm for 5 min and filtered with Whatman filter paper. Then, 1 mL of the filtrate was poured into a test tube and diluted with 5 mL of vanillin and HCl reagent prepared by an equal volume of 4% vanillin in methanol and 8% concentrated HCl in methanol. The standard stock solution was prepared by mixing 40 mg of D (+)‐catechin with 100 mL of 1% HCl solution in methanol. A standard solution (0, 12, 24, 36, 48, and 60 μg/mL) was prepared using 1% HCl in methanol. The absorbance of the standard solution and sample was measured after 20 min of incubation at 500 nm using a UV–VIS spectrophotometer (2031 Visible Spectrophotometer, Shanghai, China). The concentration of condensed tannin content of the sample was determined from a standard catechin curve and the result was expressed as mg/100 g.

(8) $$\text{Condensed tannin}\;\left(\frac{\mathrm{mg}}{100g}\right)=\frac{\left[\left(\mathrm{As}-\mathrm{Ab}\right)-\text{intercept}\right]\times 10}{\text{Slope}\times W}$$

Where: As = sample absorbance, Ab = blank absorbance, 10 = dilution factor. *W* = sample weight (g).

### 2.8 Determination of the Molar Ratio of Antinutrient to Mineral

The molar ratio of phytate to minerals (Ca, Zn, and Fe) was calculated by dividing the mole of phytate (mass of phytate/660 g/mol) to the mole of minerals (mass of Ca/M mass of Ca = 40 g/mol; mass of Zn/molar mass of Zn = 65 g/mol; mass of Fe/molar mass of Fe = 56 g/mol) (FAO 2018). Also, the oxalate to calcium molar ratio was calculated by dividing the mole of oxalate (mass of oxalate/88 g/mol) by the calcium mole. The calculated values of the molar ratios were also compared with the reported critical toxicity values (WHO/FAO 2004).

### 2.9 Statistical Analysis

The data were analyzed in triplicate using one‐way analysis of variance (ANOVA) at a *p* < 0.05 significance level using Minitab statistical software. Prior to analysis, assumptions of homogeneity of variance and normality were tested. Tukey's HSD test was used for mean separation. Results are presented as mean values ± standard error of the mean (SEM).

## 3 Results and Discussion

### 3.1 Proximate Composition

The proximate composition analysis indicated the composition of macromolecules of wild edible plant parts, which are mainly used for nutritional fact labeling for food processing industries. The total energy contents and proximate compositions evaluated on a dry matter basis for the fruit of *Mussaenda arcuata*, leaves of *Celosia trigyna* , *Pteridium aquilinum* are shown in Table 1.

**TABLE 1:** Proximate composition of selected wild edible plants in Ethiopia (g/100 g dry weight base).

WEPs samples	MC (wb)	Proximate composition						Energy (Kcal/100 g)
		MC (db) (g/100 g)	Crude Protein (g/100 g)	Crude fat (g/100 g)	Crude fiber (g/100 g)	Total ash (g/100 g)	Total CHO (g/100 g)	
*Mussaenda arcuata*	78.95^a^ ± 0.19	5.89^a^ ± 0.59	8.25^b^ ± 0.09	6.47^a^ ± 0.02	13.08^a^ ± 0.14	5.94^b^ ± 0.01	60.37^a^ ± 0.39	332.73^a^ ± 1.97
*Celosia trigyna*	85.34^b^ ± 0.52	5.92^a^ ± 0.55	29.89^a^ ± 0.54	1.53^b^ ± 0.09	11.89^b^ ± 0.89	13.53^a^ ± 0.04	37.24^b^ ± 1.24	282.32^c^ ± 3.33
*Pteridium aquilinum*	76.87^c^ ± 0.42	5.59^a^ ± 0.32	31.76^a^ ± 0.71	2.41^c^ ± 0.02	16.11^b^ ± 0.24	6.58^b^ ± 0.02	37.55^b^ ± 0.96	298.92^b^ ± 1.18
CV (%)	0.81	6.56	2.95	3.85	4.73	3.49	3.77	1.25
LSD (*p* < 0.05)	0.57	0.35	0.73	0.07	0.62	0.04	1.32	3.30

*Note:* All the values given are means of three independent measurements ± standard error (SE). Means not followed by the same superscript letters in each column of are significantly (*p* < 0.05) different from each other.

#### 3.1.1 Moisture Content

The moisture contents of the fresh WEPs ranged from 76.87 to 85.34 g/100 g. The fresh moisture contents of *Mussaenda arcuata* fruit, *Celosia trigyna* leaves, and *Pteridium aquilinum* fronds were 78.95, 85.34, and 76.87 g/100 g, respectively. The findings were consistent with previous studies that most fresh WEPs have high moisture contents, up to 90%, even though it depends on the type, structural difference, and development stage of fruits and vegetables (Vincente et al. 2014). However, the higher moisture content in fresh WEPs creates a conducive environment for the growth and development of spoilage microorganisms and enzymatic activities that destroy the quality of the product (Keyata et al. 2020). On a dry weight basis, the moisture content was determined to be 5.89 g/100 g for *M. arcuata* fruit, 5.92 g/100 g for *C. trigyna* leaves, and 5.59 g/100 g for *P. aquilinum* fronds. The observed variation in moisture content among the WEPs can be attributed to differences in plant species, anatomical parts analyzed (fruit, leaf, or frond), and environmental conditions such as soil type, humidity, and stage of maturity at harvest. Leafy vegetables, such as *Celosia trigyna* , generally exhibit higher moisture content due to their high surface area and thin cell walls, facilitating greater water retention (Aletor et al. 2002). On the contrary, fronds such as those of *Pteridium aquilinum* may have slightly lower moisture levels because of their more fibrous structure. Furthermore, moisture content is influenced by species‐specific metabolic and physiological characteristics (Odhav et al. 2007). Seasonal variation and postharvest handling can also impact moisture retention (Aberoumand 2009).

The dry weight moisture values obtained in this study align with previous findings by Yimer et al. (2023) who reported moisture contents ranging from 5.2 g/100 g in *Dioscorea praehensilis* to 6.0 g/100 g in *Solanum nigrum* in Southwestern Ethiopia. However, the values are lower than the study conducted at Eastern Wollega of Ethiopia, ranging from 9.83 g/100 g in *Dovyalis Abyssinica* to 13.10 g/100 g in *Ziziphus spina‐chris* (Jiru et al. 2023). Conversely, the values are higher than the moisture contents of *Ficus mucuso* (3.1 g/100 g) and *Mimusops kummel* (4.0/100 g), as reported in North Shewa, Dera District (Tafesse 2018).

#### 3.1.2 Crude Fat

The crude fat content of the studied wild edible plants (WEPs) showed considerable variation, ranging from 1.53 to 6.47 g/100 g on a dry weight basis. Among the three species analyzed, *Mussaenda arcuata* fruit exhibited the highest crude fat content (6.47 g/100 g), followed by *Pteridium aquilinum* fronds (2.41 g/100 g), and *Celosia trigyna* leaves (1.53 g/100 g). The relatively high fat content in *Mussaenda arcuata* suggests its potential as an energy‐rich wild fruit, especially in food‐insecure regions where dietary fats are often lacking.

The fruit of *Mussaenda arcuata* fruit had a higher crude fat content than conventionally cultivated fruits such as orange (0.2 g/100 g), banana (0.1 g/100 g), and Apple (0.006 g/100 g) (Vincente et al. 2014). It had a crude fat content comparable to *Trilepisium madagascariense* (6.1 g/100 g), a WEP consumed in Southwestern Ethiopia (Yimer et al. 2023), and *Portulaca oleracea* (6.46 g/100 g) in Pakistan (Ullah et al. 2017).

The leaves of *Celosia trigyna* and fronds of *Pteridium aquilinum* contained lower fat levels (1.53 and 2.41/100 g) respectively compared with the fruit of *Mussaenda arcuata*. However these values were within the range reported for other traditional leafy vegetables. For instance, the fat content of *C. trigyna* (1.53 g/100 g) was comparable to *Dovyalis abyssinica* (1.46 g/100 g) in Western Ethiopia (Jiru et al. 2023) and *Sisymbrium officinale* (1.51 g/100 g) in Pakistan (Shad et al. 2013). Similarly, the crude fat content of *Pteridium aquilinum* (2.41 g/100 g) was in line with that of *Haplocarpha rueppelii* (2.67 g/100 g) in Northeastern Ethiopia (Adamu et al. 2022) and *Sphenostylis stenocarpa* (2.41 g/100 g) reported in Nigeria (Sam 2019). However, the results of the present findings are lower than African spinach (3.72 g/100 g) analyzed in Nigeria (Ukom and Obi 2018).

The finding of this research indicated that the crude fat content was much higher than that of *Ficus sur* (0.12/100 g), which was conducted in the Mekdela District, South of Wollo, Ethiopia (Yiblet 2024) and lower than that found in *Rumex Abyssinicus* (young shoots) (14.07 g/100 g) that was consumed in the Tach Gaint District, Northwestern Ethiopia (Yiblet and Adamu 2023).

#### 3.1.3 Crude Protein

The crude protein contents of *Mussaenda arcuata* fruits, *Celosia trigyna* leaves, and *Pteridium aquilinum* fronds on a dry weight basis were found to be 8.25 g/100 g, 29.89 g/100 g, and 31.76 g/100 g, respectively. These values indicate that the three wild edible plants (WEPs) possess considerable protein levels. *Celosia trigyna* and *Pteridium aquilinum* significantly exceed the protein content in many commonly consumed vegetables.

The protein content of the *Mussaenda arcuata* fruit, while lower than that of the other two species, still shows nutritional promise. Its crude protein value of 8.25 g/100 g is closely comparable to that of the local pepper (8.7 g/100 g), which is widely consumed in the Bako town area of Ethiopia (Esayas et al. 2011). Additionally, this value falls within the reported range for mango fruits (6–13 g/100 g), a well‐known fruit studied in Egypt for its nutritional content (Abdalla et al. 2007). Compared to other wild edible fruits consumed in Ethiopia, *Mussaenda arcuata* has a crude protein level similar to *Cordia africana* (8.1/100 g) and higher than *Ziziphus spina‐christi* (5.3 g/100 g), both of which are traditional WEPs consumed in the Northern and Rift Valley regions (Mokria et al. 2022). These comparisons indicate that *Mussaenda arcuata* can contribute significantly to dietary protein intake, particularly in rural and nutritionally vulnerable communities.

However, the crude protein contents of *Celosia trigyna* leaves (29.89 g/100 g) and *Pteridium aquilinum* fronds (31.76 g/100 g) are remarkably high. These values are significantly higher than the crude protein content of *African spinach* (15.53 g/100 g), a leafy cultivated vegetable analyzed in Nigeria (Ukom and Obi 2018). Protein levels in these WEPs are also comparable to *Erucastrum arabicum* (30.15 g/100 g), another highly nutritious wild vegetable consumed in the Lasta district of the Amhara region in Ethiopia. Furthermore, their protein content surpasses that of *Amaranthus hybridus* seeds (23.31 g/100 g), which are widely consumed in Northeastern Ethiopia (Adamu et al. 2022).

These findings are nutritionally significant because the protein content in *Celosia trigyna* and *Pteridium aquilinum* is within the same range as that found in legume crops (20–40 g/100 g), which are typically relied upon as major plant‐based protein sources in many developing countries. Furthermore, these two species exceed the average protein content of cereal grains (10–15 g/100 g), which often serve as staple foods but generally lower in protein (Erbersdobler et al. 2017). This finding suggests that the incorporation of *Celosia trigyna* and *Pteridium aquilinum* into local diets could improve protein intake, especially in areas with limited animal protein or cultivated legumes. Overall, the high protein values observed in these WEPs support their potential role in enhancing food security and dietary diversity. Their inclusion in local food systems could benefit communities facing protein deficiency or malnutrition.

#### 3.1.4 Crude Fiber

The crude fiber content of the studied wild edible plants showed considerable variation, ranging from 11.89 g/100 g in the leaves of *Celosia trigyna* to 16.11 g/100 g in the fronds of *Pteridium aquilinum* , with *Mussaenda arcuata* fruit falling between these values. These levels meet the recommended daily allowance (RDA) for dietary fiber, which varies from 5% to 22% depending on individual dietary needs and physiological conditions (Spiller 2001). Such results affirm the high nutritional potential of these wild plants in providing fiber, a critical but often under‐consumed dietary component in many developing regions.

Compared to conventionally cultivated fruits and vegetables, the crude fiber content of the wild species studied is significantly higher. For instance, commonly consumed produce such as avocado (6.8 g/100 g), banana, apple, orange (all ~2.4 g/100 g), and tomato (1.2 g/100 g) offer substantially lower amounts of fiber (Vincente et al. 2014). This finding highlights a key nutritional advantage of underutilized wild edible plants, which are often overlooked despite their dense nutrient profiles and cultural relevance in many indigenous diets.

Furthermore, the total crude fiber in *Mussaenda arcuata* fruit and *Celosia trigyna* leaves is comparable to *Justicia ladanoides* (12.5 g/100 g), a species traditionally used as a vegetable in parts of southern Ethiopia (Getachew et al. 2013), and *Lasiurus scindicus* (12.89 g/100 g), a forage species found in Egypt (Al‐Rowaily et al. 2019). The crude fiber content of *Pteridium aquilinum* fronds is also consistent with that reported in *Portulaca quadrifida* (15.9 g/100 g) (Getachew et al. 2013) and *Eclipta alba* (16.56 g/100 g), plants consumed for their medicinal and nutritional value in Bangladesh (Rana et al. 2019).

On a broader comparative scale, the crude fiber values observed in these plants are significantly higher than those of other wild edible plants reported in the literature. For example, *Euclea racemosa* (1.53 g/100 g) (Yiblet 2024), *Cordia africana* (3.86 g/100 g) (Mokria et al. 2022), and *Dioscorea praehensilis* (8.9 g/100 g) (Yimer et al. 2023) all demonstrate much lower fiber concentrations. However, it is also important to note that some wild species, such as *Rumex abyssinicus* (34.70 g/100 g) (Yiblet and Adamu 2023) and *Erucastrum arabicum* (21.54 g/100 g) (Adamu et al. 2022), exhibit extremely high levels of crude fiber, indicating the wide variability among wild edible species depending on habitat, genotype, and plant part consumed.

From a nutritional science point of view, dietary fiber is multifaceted in promoting human health. It facilitates digestive regularity, reduces constipation, and supports the growth of the beneficial gut microbiota. Epidemiological studies have consistently shown that high‐fiber diets are associated with a reduced risk of chronic diseases such as type 2 diabetes, obesity, cardiovascular diseases, and certain types of cancer (Meyer et al. 2000; Rimm et al. 1996; Wolk et al. 1999). Furthermore, dietary fiber has been shown to help modulate blood glucose levels, improve satiety, and aid in long‐term weight control (Martins et al. 2011), making it a vital nutrient in preventive healthcare and clinical dietary interventions.

Nonetheless, while high fiber intake offers many benefits, it is important to consider that excessive fiber can affect the bioavailability of certain micronutrients and phytochemicals by forming complexes or by physical entrapment, thus reducing their absorption in the gastrointestinal tract (Palafox Carlos et al. 2011). This factor should be considered when recommending high‐fiber wild plants as part of a regular diet, especially in populations already at risk of micronutrient deficiencies.

The significant crude fiber content of *Celosia trigyna, Pteridium aquilinum*, and *Mussaenda arcuata* underscores their potential as valuable traditional foods and strategic components in nutrition‐sensitive agriculture. These plants can address fiber deficiency, improve dietary diversity, and promote overall health in rural and urban populations. Their inclusion in dietary interventions and sustainable food systems could contribute meaningfully to reducing diet‐related non‐communicable diseases and enhance food security in regions where access to commercial fruits and vegetables is limited.

#### 3.1.5 Total Ash

The leaves of *Celosia trigyna* contain a significantly higher ash content (13.53 g/100 g) than *Mussaenda arcuata* (5.94 g/100 g) and *Pteridium aquilinum* fronds (6.58 g/100 g), indicating a higher presence of minerals. This higher ash content suggests that *Celosia trigyna* could play a role in the management of micronutrient deficiencies and the improvement of health, particularly in regions where access to a variety of nutrient‐rich foods is limited. The ash content of *Mussaenda arcuata* fruit is comparable to that of the commercially cultivated Maraka Fana pepper variety (5.3 g/100 g), grown at the Bako Research Center in Ethiopia, underscoring its moderate mineral content (Esayas et al. 2011). The ash content in *Mussaenda arcuata* was similar to that of *Grewia flavescens* (5.73 g/100 g), a wild edible plant consumed in Ethiopia's East Shewa Zone (Feyssa et al. 2011).

The leaves of *Celosia trigyna* showed a consistent ash content with *Adenia ellenbeckii* (13.8 g/100 g), which was studied in southern Ethiopia (Getachew et al. 2013), highlighting its similar mineral composition. The ash content in *Pteridium aquilinum* was nearly identical to that of *Dobera glabra* (6.79 g/100 g), analyzed in the Aba'ala Woreda region of the North Afar region in Ethiopia (Tsegaye et al. 2007), further supporting its potential as a source of essential minerals.

These findings indicate that the mineral content of *Celosia trigyna, Mussaenda arcuata*, and *Pteridium aquilinum* falls within a range of values observed in other well‐studied wild edible plants (WEPs). Specifically, the ash content in these plants is higher than that of *Rhus vulgaris* (2.41 g/100 g) (Yiblet 2024) and lower than that in *Justicia flava* (25.6 g/100 g), which was consumed in southern Ethiopia (Getachew et al. 2013). These comparisons suggest that while the mineral concentrations of *Celosia trigyna, Mussaenda arcuata* and *Pteridium aquilinum* are not the highest among WEPs, they still provide valuable sources of essential minerals that can contribute to improving the overall micronutrient intake of individuals in areas with limited access to other sources of nutrition.

#### 3.1.6 Total Carbohydrate

The *Mussaenda arcuata fruit* had a high total carbohydrate (60.37 g/100 g), while the leaves of *Celosia trigyna* contained the lowest (37.24 g/100 g). Total carbohydrate in the fruit was consistent with the truth that approximately 50–80 g/100 g of fruit and vegetables are occupied by total carbohydrates (Vincente et al. 2014).

The total carbohydrate content of *Mussaenda arcuata* was consistent with the content of *Cordia Africana* (60.26 g/100 g) (Mokria et al. 2022) in Northern Ethiopia, and *Corchorus capsularis* (60.21 g/100 g) in Bangladesh (Satter et al. 2016). On the contrary, the contents of the *Celosia trigyna* leaf and the *Pteridium aquilinum* fronds were nearly comparable to the contents found in *Solanum nigrum* (38.1 g/100 g) in southern Ethiopia (Yimer et al. 2023) and similar wild edible plants in South Africa (37.23 g/100 g) (Afolayan and Jimoh 2009).

The findings of this study showed that the total carbohydrate contents of the study WEPs were found in the ranges 30.7 g/100 g in *Celosia argentea* to 83.5 g/100 g in *Gomboczianus* (Getachew et al. 2013).

### 3.2 Total Energy Contents

The study revealed that the total energy content of *Mussaenda arcuata* fruits was higher (332.73 ± 1.97 Kcal/100 g) compared to *Celosia trigyna* leaves (282.32 ± 3.33 Kcal/100 g) and *Pteridium aquilinum* fronds (298.92 ± 1.18 Kcal/100 g). These values exceed the energy content reported in a previous study conducted in Bako Town, Ethiopia, where the local pepper yielded 278.3 Kcal/100 g, as noted by Esayas et al. (2011). Similarly, the current findings surpass the energy values of some wild edible plants consumed in southern Ethiopia, such as *Solanum nigrum* (275 Kcal/100 g) and *Cleome gynandra* (276 Kcal/100 g), as reported by Yimer et al. (2023). However, the energy yields were lower than those of certain wild edible plants from North Shewa, Ethiopia, including *Ficus mucuso* (391.63 Kcal/100 g) and *Mimusops kummel* (389.83 Kcal/100 g), as documented by Tafesse (2018).

### 3.3 Mineral Content

Minerals play a key role in our body, from building strong bones to transmitting nerve impulses for a healthy and long life. The existence of a series of minerals cannot only make different hormones but also regulate a standard heartbeat (Gharibzahedi and Jafari 2017). The mineral contents of the studied wild edible plants are stated in Table 2.

**TABLE 2:** Mineral contents of selected WEPs eaten in Ethiopia (mg/100 g dry weight base).

WEPs samples	Minerals (mg/100 g)						
	K	Na	Ca	Mg	P	Fe	Zn
*Mussaenda arcuata*	108.21^a^ ± 1.30	2.5^b^ ± 0.05	62.57^b^ ± 0.59	66.60^c^ ± 0.84	85.32^b^ ± 0.47	45.59^a^ ± 0.92	4.74^b^ ± 0.14
*Celosia trigyna*	107.42^a^ ± 3.76	1.61^c^ ± 0.06	419.15^a^ ± 9.62	98.53^a^ ± 0.39	156.19^a^ ± 0.13	37.79^b^ ± 0.71	3.72^c^ ± 0.02
*Pteridium aquilinum*	105.62^a^ ± 1.31	5.67^a^ ± 0.08	31.85^c^ ± 0.98	77.29^b^ ± 0.23	44.71^c^ ± 0.49	26.25^c^ ± 0.13	5.24^a^ ± 0.06
CV (%)	3.94	3.43	3.64	1.14	1.00	2.53	2.69
LSD (*p* < 0.05)	3.41	0.09	7.91	0.78	0.57	1.24	0.12

*Note:* All the given values are means of three independent measurements ± standard error (SE). Means not followed by the same superscript letters in each column of WEPs are significantly (*p* < 0.05) different from each other.

#### 3.3.1 Potassium

Inadequate potassium intake has long been associated with increased mortality (Wang et al. 2014) and higher blood pressure (Mccarron and Reusser 2001). The potassium content in the finding ranged from 105.62 mg/100 g in the *Pteridium aquilinum* frond to 108.21 mg/100 g in the *Mussaenda arcuata* fruit. They contain comparative potassium content with *Gardenia erubescens* (107.54 mg/100 g) that was analyzed in Western Ethiopia (Jiru et al. 2023). They also contain far lower content than the contents in *Balanites aegeyptica* (2539.04 g/100 g) that was conducted in the Northern and Rift Valley region of Ethiopia (Mokria et al. 2022), and higher than in the grain of *Amaranthus hybridus* (14.4 mg/100 g) that was examined in Northeast Ethiopia (Adamu et al. 2022), and *Vernonia amygdalina* (3.62 g/100 g) that was analyzed in Cameroon (Mih et al. 2017).

#### 3.3.2 Sodium

The dietary sodium contents in the studied wild edible plants were found in the range of 1.61 mg/100 g in *Celosia trigyna* to 5.67 mg/100 g in *Pteridium aquilinum fronds*, which were lower than the sodium content in *Ziziphus spina‐christi* (6.01 mg/100 g) that was studied in Western Ethiopia (Jiru et al. 2023). The low sodium content in foods is vital for better health, particularly for people susceptible to high blood pressure. Reducing dietary sodium intake not only decreases blood pressure levels and the incidence of hypertension, but is also associated with a reduction in cardiovascular morbidity and mortality (Whelton and He 2014).

#### 3.3.3 Calcium

Calcium is an essential mineral in the human diet for developing strong bones and teeth, controlling muscle contractions, and transmitting nerve impulses throughout the body (Power et al. 1999). The finding showed that the dietary calcium contents of the study wild edible plants were 419.15, 62.57, and 31.85 mg/100 g for leaves of *Celosia trigyna* , the fruit of *Mussaenda arcuata*, and the fiddlehead of *Pteridium aquilinum*, respectively.

The calcium content in the findings was found in the ranges conducted in Southern Ethiopia by Yimer et al. (2023) from 3.7 mg/100 g in *Dioscorea praehensilis* to 594.8 mg/100 g in *Cleome gynandra* . The findings were far lower than the content of calcium found in *Justicia ladanoides* (6177 mg/100 g), which was a calcium‐rich wild edible plant consumed in Southern Ethiopia (Getachew et al. 2013), and higher than the content in *Oxytenanthera abyssinica* (24.49 mg/100 g) conducted in the Benishangul Gumuz region of Ethiopia (Andinet and Getachew 2016).

#### 3.3.4 Magnesium

The magnesium contents of the study wild edible plants vary from 66.6 mg/100 g in *Mussaenda arcuata* fruit to 98.53 mg/100 g in *Celosia trigyna* leaf. They contain more dietary magnesium than the content in *Erucastrum arabicum* leaves (56.65 mg/100 g) that were explored in Northeastern Ethiopia (Adamu et al. 2022). On the contrary, they contain less magnesium than *Amaranthus graecizans* (2049 mg/100 g), a magnesium‐rich wild edible plant eaten in Southern Ethiopia (Getachew et al. 2013). The higher magnesium contents in this study are essential for maintaining electrical potential in nerves and membranes and for the normal metabolism of calcium and phosphorus (Afolayan and Jimoh 2009). It has also been linked to various enzymatic reactions, including nutrient oxidative metabolism, cell constituent synthesis, nerve impulse transmission, body temperature regulation, detoxification, energy production, and the development of strong bones and teeth (Soetan et al. 2010).

#### 3.3.5 Phosphorus

Inorganic phosphate is essential for skeletal mineralization and multiple cellular functions, including glycolysis, gluconeogenesis, DNA synthesis, RNA synthesis, cellular protein phosphorylation, phospholipid synthesis, and intracellular regulatory roles (Dimeglio et al. 2000). The Phosphorus contents in *Celosia trigyna* leaf, *Mussaenda arcuata* fruit, and *Pteridium aquilinum* fiddlehead were 156.19, 85.32, and 44.71 mg/100 g, respectively. The phosphorus content in the fruit of *Mussaenda arcuata* and leaves of *Celosia trigyna* was higher than the content in the previous study conducted by Jiru et al. (2023) in Western Ethiopia in *Ziziphus spina‐christi* (55.76 mg/100 g) but lower than in *Dovyalis abyssinica* (168 mg/100 g). The findings from the study were also higher than the content in *Dioscorea abyssinica* (40.68 g/100 g) conducted at Benishangul Gumuz region of Ethiopia (Andinet and Getachew 2016) and in *Gnetum africanum* (0.19 mg/100 g) at Cameroon (Mih et al. 2017). The high phosphorus content found in edible plants is essential for body development and the formation of bone structure (Onwordi et al. 2009).

#### 3.3.6 Iron

Iron is required for many human metabolic processes, including DNA synthesis, electron transport, oxygen transport, and vital enzymatic activities. Regulation of serum iron involves complex mechanisms and is essential to minimize the impact of iron deficiency and overload (Li et al. 2020). The iron contents of the study wild edible plants were found in ranges 26.25 mg/100 g from *Pteridium aquilinum* fronds and 45.59 mg/100 g from *Mussaenda arcuata* fruits. The findings are higher than the previous study by Yimer et al. (2023) in Southern Ethiopia, which found in *Cleome gynandra* (21.7 mg/100 g) and *Solanum nigrum* (26.9 mg/100 g) and significantly higher than the content in *Trilepisium madagascariense* (0.8 mg/100 g) and *Gnetum africanum* (0.02 mg/100 g) that were explored in Cameroon (Mih et al. 2017). The finding was also higher than the iron content in *Vitex negundo* (23.40 g/100 g) examined in Bangladesh (Rana et al. 2019). The iron content in *Mussaenda arcuata* fruits and *Celosia trigyna* leaves was nearly comparable to that in *Rhus natalensis* (41 mg/100 g), as explored in Northern Ethiopia (Yiblet 2024). The study from the findings reveals that the consumption of these wild edible plants provides a dietary iron that can participate in a wide variety of metabolic processes, including oxygen transport, deoxyribonucleic acid (DNA) synthesis, and electron transport (Abbaspour et al. 2014).

#### 3.3.7 Zinc

Zinc is a pervasive and versatile microelement that plays a catalytic or structural role in over 300 enzymes involved in digestion (carboxypeptidase, liver alcohol dehydrogenase, carbonic anhydrase), metabolism, reproduction, and wound healing (Prasad 2017). The dietary zinc contents of the study wild edible plants in Leaves of *Celosia trigyna* , *Mussaenda arcuata* fruits, and *Pteridium aquilinum* fronds were 3.72, 4.74, and 5.24 mg/100 g, respectively. The findings were comparable to the study in Southern Ethiopia, whose zinc contents ranged from 3.7 mg/100 g in *Solanum nigrum* to 5.5 mg/100 g in *Cleome gynandra (*Yimer et al. 2023). They contained significantly higher than the content in *Ziziphus spania‐chrsti* (0.35 mg/100 g) that was explored in Northern Ethiopia (Mokria et al. 2022). Consuming wild edible plants rich in dietary zinc could play a great role in the immune system, cell division, cell growth, wound healing, and breakdown of carbohydrates (Roohani et al. 2013).

### 3.4 Mineral Ratio

The mineral ratio with critical limit values is shown in Table 3. The ratios of Na/K for the studied wild edible plants were between 0.026 and 0.091. It was reported by Ijarotimi et al. (2013) that the value of a ratio of Na/K less than one was recommended for healthy foods. The results of this study reveal that all the studied wild edible plants are in the recommended range and safe for consumption. The Na: K ratio reduction below one is critical for reducing cardiovascular diseases and hypertension (Binia et al. 2015). The results suggest that the regular consumption of *Mussaenda arcuata* fruit, *Celosia trigyna* leaves, and *Pteridium aquilinum* fronds may contribute to dietary strategies aimed at reducing hypertension. However, further clinical studies are needed to confirm this potential benefit.

**TABLE 3:** Mineral ratio in selected wild edible plants in Ethiopia.

WEPs	Mineral ratio			
	(Na: K)^1^	(Ca: P)^2^	(Ca: K)^3^	(Fe:Zn)^4^
*Mussaende arcuata*	0.039^b^ ± 0.001	0.57^c^ ± 0.008	0.56^b^ ± 0.004	11.19^a^ ± 0.43
*Celosia trigyna*	0.026^c^ ± 0.001	2.08^a^ ± 0.05	3.81^a^ ± 0.05	11.78^a^ ± 0.29
*Pteridium aquilinum*	0.091^a^ ± 0.002	0.55^b^ ± 0.02	0.29^b^ ± 0.008	5.81^b^ ± 0.038
Standard values	< 0.5	> 0.5	< 4	> 2

*Note:* All the given values are means of three independent measurements ± standard error (SE). Means not followed by the same superscript letters in each column of WEPs are significantly (*p* < 0.05) different from each other.

The calcium‐to‐phosphorus (Ca/P) ratio is an important nutritional parameter influencing calcium absorption and bone health. A Ca/P ratio > 0.5 has been recommended for healthy diets, as it facilitates optimal calcium absorption in the small intestine (Jacob et al. 2015). Diets with low Ca/P ratios may lead to impaired calcium utilization and potential bone demineralization. In this study, all the analyzed wild edible plants exhibited Ca/P ratios above the recommended threshold, ranging from 0.55 to 2.08. This favorable ratio supports efficient calcium absorption, which is particularly beneficial for growing children who require higher intakes of calcium and phosphorus for the proper development of bones and teeth (Ijarotimi et al. 2013).

The calcium‐to‐potassium (Ca/K) ratio is a critical nutritional factor that can influence various physiological processes, including electrolyte balance and endocrine function. According to Watts (2010), an ideal Ca/K ratio is approximately 4:1, with an acceptable range between 2.2 and 6.2. Maintaining this balance is important for optimal metabolic function, as significant deviations may impact cellular activities, including enzyme regulation and hormone activity. Notably, an elevated Ca/K ratio has been linked to altered thyroid function, potentially inhibiting the activity of thyroid hormones (Pérès et al. 2001). In this study, all the wild edible plants analyzed exhibited Ca/K ratios below the maximum acceptable limit (i.e., below 4), suggesting that their consumption may support a balanced mineral profile conducive to healthy thyroid hormone activity.

The iron‐to‐zinc (Fe/Zn) ratio is an important indicator of the potential interaction between these two essential trace elements during intestinal absorption. High dietary iron levels can inhibit zinc absorption, particularly when present in soluble forms or in high molar excess. However, Pérès et al. (2001) reported that zinc absorption is not significantly impaired when the Fe/Zn molar ratio remains below 2:1, and that increasing the ratio to between 5:1 and 10:1 does not result in further inhibition. In the present study, the Fe/Zn ratios of the wild edible plants ranged from 5.81 to 11.78, suggesting that while the ratios are above the initial 2:1 threshold, they remain within a range that has not been shown to negatively affect zinc bioavailability. This implies that the iron content in the fruit of *Mussaenda arcuata*, the leaves of *Celosia trigyna* , and the fronds of *Pteridium aquilinum* is unlikely to interfere significantly with zinc absorption. Therefore, the consumption of these wild edible plants may contribute meaningfully to addressing micronutrient deficiencies, particularly iron and zinc, which are crucial for immune function, growth, and cognitive development (Dangoggo et al. 2011).

### 3.5 Anti‐Nutritional Factor Contents

Anti‐nutritional factors reduce the maximum utilization of nutrients, especially proteins, vitamins, and minerals (Ugwu and Oranye 2006). The anti‐nutritional factor contents of the studied wild edible plants are shown in Table 4.

**TABLE 4:** Anti‐nutritional contents of selected WEPs in Ethiopia.

WEPs samples	Anti‐nutritional contents		
	Condensed tannin (mg CE/100 g)	Oxalate (mg/100 g)	Phytate (mg/100 g)
*Mussaenda arcuata*	25.95^a^ ± 1.44	48.42^a^ ± 0.52	197.74^a^ ± 3.50
*Celosia trigyna*	22.07^b^ ± 0.31	29.93^a^ ± 0.46	172.11^b^ ± 1.42
*Pteridium aquilinum*	6.73^c^ ± 0.13	13.35^c^ ± 0.25	103.67^c^ ± 2.43
CV (%)	5.14	2.55	2.86
LSD (*p* < 0.05)	1.21	0.61	3.67

*Note:* All the given values are means of three independent measurements ± standard error (SE). Means not followed by the same superscript letters in each column of WEPs are significantly (*p* < 0.05) different from each other.

#### 3.5.1 Oxalate

The total oxalate content of the study wild edible plants was found in 13.35 mg/100 g for fronds of *Pteridium aquilinum* to 48.42 mg/100 g from the fruit of *Mussaenda arcuata*. The findings were found to be higher than the study conducted in the Northern part of Ethiopia in *Rhus vulgaris* (0.52 mg/100 g) (Yiblet 2024) and lower than the content in *Solanum nigrum* (443.9 mg/100 g) investigated in Southern Ethiopia (Yimer et al. 2023). The findings from this study were below the maximum acceptable level for oxalate (50 mg/100 g); hence, consuming these wild edible plants did not cause any toxicity to human health (Kasimala et al. 2018).

#### 3.5.2 Phytate

The presence of Phytate in food products decreases the digestibility of amino acids. It hinders the absorption of calcium, magnesium, phosphorus, zinc, and iron by complexing with them, leading to the body's unavailability of these essential minerals (Silva et al. 2021). The phytate contents in the study wild edible plants were high in *Mussaenda arcuata* (197.74 mg/100 g), followed by the content in *Celosia trigyna* leaf (172.11 mg/100 g), and low in *Pteridium aquilinum* (103.67 mg/100 g).

The findings were nearly comparable to and lower than the content in *Solanum nigrum* (233.3 mg/100 g) conducted in Southern Ethiopia (Yimer et al. 2023). The findings from this study showed that the phytate contents from the studied wild edible plants were below the maximum tolerable level (200 mg/100 g) (Hurrell 2004), which may not impair protein digestibility and the bioavailability of zinc, calcium, and iron (Oghbaei and Prakash 2016).

#### 3.5.3 Condensed Tannin

Due to its astringent properties, tannin has been demonstrated to decrease food palatability, impede the activity of certain enzymes, and complex with proteins to reduce their solubility and digestibility (Zhang et al. 2023). They also chelate metals like Fe and Zn to hinder their bioavailability (Sam 2019). The condensed tannin contents in the Fruit of *Mussaenda arcuata*, *Celosia trigyna* leaf, and *Pteridium aquilinum* fronds were 25.95, 22.07, and 6.73 mg CE/100 g, respectively. The contents in Fruit of *Mussaenda arcuata* and the leaf of *Celosia trigyna* were nearly comparable to the contents in *Trilepisium madagascariense* (28.9 mg CE/100 g) in Southern Ethiopia described by Yimer et al. (2023) and *Cyperus conglomeratus* (26.1 mg CE/100 g) in Egypt (Al‐Rowaily et al. 2019), but far lower than that was found in *Cleome gynandra* (329.9 mg CE/100 g) studied in Southern Ethiopia (Yimer et al. 2023). The condensed tannin from the fronds of *Pteridium aquilinum* was comparable with the content in *Balanites aegyptiaca* (6.12 mg CE/100 g), which was conducted in Central Ethiopia (Tafesse 2018) and higher than *Rhus natalensis* (0.23 mg CE/100 g), which was examined at Northern Ethiopia (Yiblet 2024). The tannin contents of the study wild edible plants considered in this study were below the toxicity level of daily intake (560 mg CE/100 g) stated by Fekadu et al. (2013).

### 3.6 Molar Ratio and Bioavailability of Minerals

Anti‐nutritional factors like oxalate and phytate in edible parts of plants interfere with the absorption and bioavailability of some essential minerals, such as calcium, zinc, and iron, which can be evaluated by calculating their molar ratios (Šimić et al. 2009). The calculated molar ratios of antinutrient: mineral of *Mussaenda arcuata* fruit, *Celosia trigyna* leaves, and *Pteridium aquilinum* fronds, with the reported critical toxicity level values for each ratio, are shown in Table 5.

**TABLE 5:** Molar ratios of anti‐nutritional factors to minerals in selected WEPs in Ethiopia.

WEPs	Molar ratios of anti‐nutritional factors to minerals				
	(Phytate: Ca)^1^	(Phytate: Fe)^2^	(Phytate: Zn)^3^	(Oxalate: Ca)^4^	(Phytate*Ca: Zn)^5^
*Mussaenda arcuata*	0.192^a^ ± 0.004	0.37^a^ ± 0.07	4.11^b^ ± 0.10	0.35^a^ ± 0.001	6.43^b^ ± 0.097
*Celosia trigyna*	0.025^b^ ± 0.001	0.39^a^ ± 0.003	4.55^a^ ± 0.066	0.03^c^ ± 0.001	47.7^a^ ± 0.1
*Pteridium aquilinum*	0.198^a^ ± 0.011	0.33^b^ ± 0.009	1.95^c^ ± 0.063	0.20^b^ ± 0.009	1.54^c^ ± 0.003
Standard values	< 0.24	> 0.15	< 10	< 1	< 0.5

*Note:* All the given values are means of three independent measurements ± standard error (SE). Means not followed by the same superscript letters in each column of WEPs are significantly (*p* < 0.05) different from each other.

The presence of phytate in edible parts of plants decreases calcium absorption. It was reported by Woldegiorgis et al. (2015) that the critical molar ratio of phytate: calcium that indicates good calcium bioavailability is < 0.24. The molar ratios of phytate: Ca from this finding ranged from 0.025 to 0.198. The phytate to calcium molar ratio of all the studied wild edible plants was below the critical molar ratio limit (< 0.24), implying that the absorption of calcium in all the studied wild edible plants is not adversely affected by phytate.

According to Hurrell (2004), a phytate‐to‐iron molar ratio below 0.4 is considered better for iron absorption. In the present study, the wild edible plants (WEPs) exhibited phytate: Fe molar ratios ranging from 0.33 to 0.39, all within the recommended threshold. These findings suggest that the phytate content in the studied WEPs is unlikely to inhibit iron bioavailability, indicating their potential as good dietary sources of bioavailable iron.

The molar ratio of Phytate: Zn of less than 10, which is sufficient for the bioavailability of zinc. However, the problem is encountered when older than 15 (Morris and Ellis 1989). It was reported by Brown et al. (2001) that there is a correlation of phytic acid to zinc molar ratio showing ≥ 15, 5–15, and < 15 with low (10%–15%), moderate (30%–35%), and high (50%–55%), zinc bioavailability respectively. In this regard, the values in fruits of *Mussaenda arcuata*, *Celosia trigyna* leaves, and *Pteridium aquilinum* fronds were below 10, indicating high Zinc bioavailability.

The molar ratio of oxalate: Ca in the studied wild edible plants ranged from 0.03 to 0.35. It was reported by WHO (2003) that the availability of dietary calcium in plant foods was limited when the oxalate: Ca molar ratio was higher than 1. The oxalate: Ca molar ratios of all the studied wild edible plants were below the critical level of one (< 1), which implies that the oxalate in the edible plant may not adversely affect the bioavailability of dietary calcium.

A molar ratio of Phytate*Ca: Zn value > 200 might negatively affect the bioavailability of Zinc in food products (Castro‐Alba et al. 2019). The values obtained from this study (1.54–47.7) were far lower than the indicated standard value and, hence, are suitable for the bioavailability of Zinc.

## 4 Conclusion

This study presented nutritional composition, anti‐nutritional factor contents, mineral composition, and their bioavailability in fruits of *Mussaenda arcuata* collected from Konta special woreda, and leaves of *Celosia trigyna* and fronds of *Pteridium aquilinum* collected from Metu Woreda of the Southwestern parts of Ethiopia. The results showed that wild edible plants are potential sources of nutrients and minerals essential for the human diet and health. The nutritional values of the studied wild edible plants were comparable and higher in some instances, with reported figures for popular exotic and widely cultivated fruit and vegetable species implying their potential utilization as food and nutrition supplements and complementing conventional crops. They contained appreciable levels of protein, carbohydrates, energy, dietary fiber, and minerals, with relatively low levels of anti‐nutritional factors, ensuring that mineral bioavailability was not significantly impaired. The molar ratios of anti‐nutritional factors to minerals were within acceptable safety thresholds, indicating suitability for consumption. Notably, the leaves of *Celosia trigyna* and fronds of *Pteridium aquilinum* were particularly rich in protein, surpassing levels typically found in conventional fruits and vegetables, and consistent with those in legume crops, making them especially beneficial for addressing protein needs in growing children. Therefore, promoting the consumption of these wild edible plants can contribute significantly to combating protein‐energy malnutrition and mineral micronutrient deficiencies in resource‐limited settings.

## Author Contributions

**Tamene Daba Rumicha:** conceptualization (equal), data curation (equal), formal analysis (equal), methodology (equal), project administration (equal), software (equal), validation (equal), visualization (equal), writing – original draft (equal), writing – review and editing (equal). **Sirawdink Fikreyesus Forsido:** conceptualization (equal), data curation (equal), formal analysis (equal), funding acquisition (equal), investigation (equal), methodology (equal), project administration (equal), supervision (equal), validation (equal), writing – original draft (equal), writing – review and editing (equal). **Yetenayet Bekele Tola:** conceptualization (equal), data curation (equal), formal analysis (equal), investigation (equal), resources (equal), software (equal), supervision (equal), validation (equal), writing – original draft (equal), writing – review and editing (equal). **Abebe Yimer:** data curation (equal), formal analysis (equal), methodology (equal), project administration (equal), supervision (equal), validation (equal), writing – original draft (equal). **Chala G. Kuyu:** conceptualization (equal), formal analysis (equal), methodology (equal), project administration (equal), resources (equal), supervision (equal), validation (equal), writing – original draft (equal). **Tilahun A. Teka:** conceptualization (equal), formal analysis (equal), investigation (equal), methodology (equal), supervision (equal), validation (equal), writing – original draft (equal). **Amira Gidi:** formal analysis (equal), methodology (equal), validation (equal), writing – original draft (equal).

## Conflicts of Interest

The authors declare no conflicts of interest.

## Data Availability

Data sets to support this are available upon reasonable request from the author.
